# Long-term homeostasis in microbial consortia via auxotrophic cross-feeding

**DOI:** 10.1101/2025.01.08.631749

**Published:** 2025-01-08

**Authors:** Nicolas E. Grandel, Amanda M. Alexander, Xiao Peng, Caroline Palamountain, Razan N. Alnahhas, Andrew J. Hirning, Krešimir Josić, Matthew R. Bennett

## Abstract

Synthetic microbial consortia are collections of multiple strains or species of engineered organisms living in a shared ecosystem. Because they can separate metabolic tasks among different strains, synthetic microbial consortia have myriad applications in developing biomaterials, biomanufacturing, and biotherapeutics. However, synthetic consortia often require burdensome control mechanisms to ensure that the members of the community remain at the correct proportions. This is especially true in continuous culture systems in which slight differences in growth rates can lead to extinctions. Here, we present a simple method for controlling consortia proportions using cross-feeding in continuous auxotrophic co-culture. We use mutually auxotrophic *E. coli* with different essential gene deletions and regulate the growth rates of members of the consortium via cross-feeding of the missing nutrients in each strain. We demonstrate precise regulation of the co-culture steady-state ratio by exogenous addition of the missing nutrients. We also model the co-culture’s behavior using a system of ordinary differential equations that enable us to predict its response to changes in nutrient concentrations. Our work provides a powerful tool for consortia proportion control with minimal metabolic costs to the constituent strains.

## Full Text Availability

The license terms selected by the author(s) for this preprint version do not permit archiving in PMC. The full text is available from the preprint server.

